# Supplementary material for: Inverse Probability of Treatment Weighting in 5-Year Quality-of-Life Comparison among Three Surgical Procedures for Hepatocellular Carcinoma
Source: Cancers (Basel). 2022 Dec 30;15(1):252. doi: 10.3390/cancers15010252 (PMC9818414; doi:10.3390/cancers15010252)
Supplement: Supplementary file 1 [file cancers-15-00252-s001.zip › cancers-2096974-supplementary.pdf]

Table S1. Differences and 95% confidence intervals in effect sizes for each FACT-Hep and EORTC QLQ-C30 subscale scores in hepatocellular carcinoma patients compared among three surgery types at different time points*.

	Laparoscopic –open	Robotic –open	Robotic–laparoscopic	Laparoscopic –open	Robotic –open	Robotic–laparoscopic	Laparoscopic –open	Robotic –open	Robotic–laparoscopic
Subscale	T2 vs T1	T2 vs T1	T2 vs T1	T3 vs T2	T3 vs T2	T3 vs T2	T4 vs T3	T4 vs T3	T4 vs T3
					FACT-Hep				
PWB	0.29(0.23, 0.35)	-0.03(-0.07, 0.01)	-0.32(-0.37, -0.27)	0.87(0.80, 0.94)	0.91(0.83, 0.99)	0.04(0.01, 0.07)	0.16(0.08, 0.24)	-0.01(-0.04, 0.02)	-0.17(-0.23, -0.11)
SWB	-0.02(-0.05, 0.02)	-0.03(-0.07, 0.01)	-0.01(-0.04, 0.02)	-0.25(-0.32, -0.18)	-0.24(-0.31, -0.17)	0.01(-0.04, 0.06)	1.05(0.90, 1.14)	1.28(1.17, 1.39)	0.23(0.13, 0.33)
EWB	0.51(0.44, 0.58)	0.33(0.27, 0.39)	-0.18(-0.24, -0.12)	0.37(0.29, 0.45)	0.48(0.40, 0.56)	0.11(0.04, 0.18)	0.41(0.31, 0.51)	0.39(0.30, 0.48)	-0.02(-0.10, 0.06)
FWB	0.31(0.25, 0.37)	0.16(0.09, 0.23)	-0.17(-0.24, -0.10)	0.50(0.40, 0.61)	0.30(0.22, 0.38)	-0.20(-0.26, -0.14)	0.45(0.34, 0.56)	0.15(0.07, 0.23)	-0.30(-0.41, -0.29)
HCS	0.08(0.02, 0.14)	0.25(0.17, 0.33)	-0.33(-0.41, -0.24)	0.86(0.74, 0.98)	0.89(0.77, 1.01)	0.03(-0.02, 0.08)	0.27(0.20, 0.34)	0.24(0.16, 0.32)	-0.03(-0.09, 0.02)
FACT-G	0.32(0.20, 0.44)	0.47(0.35, 0.59)	0.15(0.05, 0.25)	0.27(0.16, 0.38)	0.28(0.20, 0.36)	0.01(-0.05, 0.06)	0.13(0.07, 0.21)	0.12(0.05, 0.19)	-0.01(-0.07, 0.05)
Total	0.48(0.37, 0.59)	0.45(0.34, 0.56)	-0.03(-0.13, 0.07)	0.20(0.10, 0.31)	0.30(0.20, 0.40)	0.10(-0.01, 0.21)	0.12(0.02, 0.22)	0.21(0.11, 0.32)	0.09(-0.02, 0.20)
					EORTC-QLQ-C30				
QL	0.12(0.04, 0.20)	0.01(-0.04, 0.05)	-0.12(-0.17, -0.05)	0.10(0.04, 0.16)	0.40(0.34, 0.46)	0.30(0.24, 0.36)	0.32(0.20, 0.43)	0.33(0.22, 0.44)	0.01(-0.09, 0.11)
PF	-0.34(-0.44, -0.24)	-0.63(-0.74, -0.52)	-0.29(-0.39, -0.19)	-0.13(-0.21, -0.07)	-0.03(-0.09, 0.03)	0.10(0.04, 0.16)	0.39(0.28, 0.50)	0.51(0.39, 0.63)	0.12(0.01, 0.23)
RF	0.41(0.30, 0.52)	-0.19(-0.29, -0.09)	-0.60(-0.70, -0.50)	0.12(0.04, 0.20)	0.15(0.08, 0.22)	0.03(-0.05, 0.11)	0.20(0.13, 0.27)	0.24(0.16, 0.32)	0.04(-0.04, 0.12)
EF	0.09(0.04, 0.14)	-0.05(-0.09, -0.01)	-0.14(-0.19, -0.09)	0.09(0.03, 0.15)	-0.03(-0.08, 0.02)	-0.12(-0.17, -0.07)	0.18(0.10, 0.26)	0.35(0.27, 0.43)	0.17(0.10, 0.24)
CF	-0.14(-0.19, -0.09)	-0.24(-0.30, -0.18)	-0.10(-0.16, -0.04)	0.17(0.08, 0.23)	-0.02(-0.08, 0.04)	-0.19(-0.26, -0.12)	0.42(0.30, 0.54)	0.34(0.22, 0.46)	-0.08(-0.15, -0.01)
SF	0.05(0.01, 0.09)	-0.14(-0.18, -0.10)	-0.19(-0.24, -0.14)	0.07(0.01, 0.13)	-0.02(-0.08, 0.04)	-0.09(-0.17, -0.01)	0.64(0.52, 0.76)	0.78(0.56, 1.01)	0.14(0.04, 0.24)
FA	-0.17(-0.25, -0.09)	0.33(0.25, 0.41)	0.50(0.40, 0.61)	-0.09(-0.14, -0.04)	-0.24(-0.32, -0.16)	-0.15(-0.22, -0.08)	-0.04(-0.12, 0.04)	-0.28(-0.38, -0.18)	-0.24(-0.34, -0.14)
NV	-0.33(-0.43, -0.23)	-0.28(-0.39, -0.17)	0.05(-0.05, 0.15)	0.32(0.20, 0.44)	-0.63(-0.77, -0.49)	-0.31(-0.43, -0.19)	-0.81(-0.93, -0.79)	0.08(-0.03, 0.19)	0.89(0.75, 1.03)
PA	0.30(0.20, 0.41)	0.11(0.01, 0.22)	-0.19(-0.27, -0.11)	0.07(0.01, 0.13)	0.01(-0.05, 0.07)	-0.07(-0.16, 0.02)	-0.35(-0.47, -0.23)	-0.23(-0.35, -0.11)	0.12(0.01, 0.23)
DY	0.29(0.18, 0.40)	0.21(0.09, 0.33)	-0.08(-0.16, 0.01)	-0.21(-0.32, -0.10)	-0.13(-0.24, -0.02)	0.08(-0.03, 0.19)	-0.24(-0.35, -0.13)	-0.23(-0.33, -0.13)	0.01(-0.09, 0.11)
SL	0.10(0.01, 0.19)	0.01(-0.08, 0.10)	-0.09(-0.17, -0.01)	-0.22(-0.33, -0.11)	-0.27(-0.38, -0.16)	-0.05(-0.16, 0.06)	0.08(0.01, 0.17)	0.11(0.01, 0.22)	0.03(-0.06, 0.12)
AP	0.38(0.24, 0.52)	0.33(0.20, 0.46)	-0.05(-0.12, 0.02)	-0.03(-0.09, 0.03)	-0.15(-0.23, -0.07)	-0.12(-0.22, -0.02)	-0.16(-0.24, -0.08)	-0.21(-0.29, -0.13)	-0.05(-0.11, 0.01)
CO	0.18(0.06, 0.30)	0.36(0.24, 0.48)	0.16(0.06, 0.26)	-0.02(-0.08, 0.04)	-0.13(-0.20, -0.07)	-0.11(-0.18, -0.04)	-0.10(-0.18, -0.02)	-0.06(-0.13, 0.01)	0.04(-0.03, 0.11)
DI	0.14(0.03, 0.25)	0.14(0.03, 0.26)	0.01(-0.04, 0.06)	-0.06(-0.11, -0.01)	0.01(-0.04, 0.06)	0.06(0.01, 0.11)	-0.06(-0.14, 0.02)	-0.06(-0.13, 0.02)	0.01(-0.04, 0.06)
FI	0.03(-0.01, 0.07)	0.03(-0.01, 0.07)	0.01(-0.03, 0.05)	0.01(-0.04, 0.06)	-0.07(-0.13, -0.01)	-0.08(-0.14, -0.02)	-0.16(-0.23, -0.10)	-0.38(-0.50, -0.26)	-0.22(-0.32, -0.12)

FACT-Hep, Functional Assessment of Cancer Therapy- Hepatobiliary; EORTC QLQ-C30, European Organization for Research and Treatment of Cancer- Quality of Life Questionnaire- Cancer 30; PWB, physical well-being; SWB, social/family well-being; EWB, emotional well-being; FWB, functional well-being; HCS, hepatobiliary cancer subscale; QL, global health status; PF, physical functioning; RF, role functioning; EF, emotional functioning; CF, cognitive functioning; SF, social functioning; FA, fatigue; NV, nausea and vomiting; PA, pain; DY, dyspnea; SL, insomnia; AP, appetite loss; CO, constipation; DI, diarrhea; FI, financial difficulties. *T1: preoperative; T2: postoperative 6-month; T3: postoperative 2-year; T4: postoperative 5-year survey.
